# A snapshot of a representative Brazilian state of illegal mining in indigenous areas during the era of malaria elimination

**DOI:** 10.1590/0102-311XEN224023

**Published:** 2024-07-29

**Authors:** Jacqueline de Aguiar Barros, Fabiana Granja, Daniel da Silva e Silva, Arthur Camurça Citó, Cássio Peterka, Maria de Fátima Ferreira-da-Cruz

**Affiliations:** 1 Coordenação Geral de Vigilância em Saúde, Secretaria de Estado da Saúde de Roraima, Boa Vista, Brasil.; 2 Programa de Pós-graduação em Biodiversidade e Biotecnologia da Rede BIONORTE, Boa Vista, Brasil.; 3 Centro de Estudos da Biodiversidade, Universidade Federal de Roraima, Boa Vista, Brasil.; 4 Núcleo de Apoio à Pesquisa em Roraima, Instituto Nacional de Pesquisas da Amazônia, Boa Vista, Brasil.; 5 Superintendência de Vigilância em Saúde do Amapá, Macapá, Brasil.; 6 Instituto Oswaldo Cruz, Fundação Oswaldo Cruz, Rio de Janeiro, Brasil.; 7 Centro de Pesquisa, Diagnóstico e Treinamento em Malária, Fundação Oswaldo Cruz, Rio de Janeiro, Brasil.

**Keywords:** Mining, Indigenous Peoples, Amazon, Malaria, Mineração, Povos Indígenas, Amazônia, Malária, Minería, Pueblos Indígenas, Amazonia, Malaria

## Abstract

Malaria is a public health problem and the cases diagnosed in the capital of Roraima, Brazil, show potential to characterize the burden of the disease in the state. This study aimed to describe the epidemiological, clinical, and laboratory aspects of malaria cases diagnosed in Boa Vista. For this purpose, a descriptive, cross-sectional study was conducted in two health units in the city, with individuals diagnosed and who agreed to respond the questionnaire. Of the total of 206 participants, characterized as men, mixed-race, and young, 96% (198) reported participating in illegal mining activity. Among the group of miners, 66% (131) came from other states of Brazil or other countries. The mines were mainly located in the Yanomami territory in Roraima. *Plasmodium vivax* infection occurred in 74% (153) of participants. In the miner’s group, hospitalizations for severe malaria, previous malaria attacks, and delays in treatment after the onset of symptoms were reported. Although 73% (145) of miners reported knowing how malaria was transmitted, only 54% (107) used mosquito nets or repellents. The use of Artecom and chloroquine by miners is not for the complete treatment but only to relieve symptoms for returning to gold mines, highlighting the importance of molecular surveillance to antimalarial resistance. Indigenous peoples are considered vulnerable to malaria and miners promotes the increase of malaria in Roraima Indigenous Lands. Therefore, access to diagnosis and treatment in Indigenous areas invaded by miners is imperative to confront this disease that ravages Indigenous communities and threatens public health on a large scale to achieve the goal of eliminating malaria in the state.

## Resumo

A malária é um problema de saúde pública e os casos diagnosticados na capital de Roraima, Brasil, têm potencial para caracterizar a carga da doença em todo o estado. O objetivo deste estudo foi descrever os aspectos epidemiológicos, clínicos e laboratoriais dos casos de malária diagnosticados em Boa Vista. Para tanto, foi realizado um estudo descritivo e transversal em duas unidades de saúde do município, com indivíduos diagnosticados com malária e que concordaram em responder ao questionário. De 206 participantes, caracterizados como homens, pardos e jovens, 96% (198) relataram atividade de garimpo ilegal. Entre garimpeiros, 66% (131) vieram de outros estados do Brasil ou de outros países. As minas estavam localizadas principalmente no território Yanomami, em Roraima. A infecção por *Plasmodium vivax* ocorreu em 74% (153) dos participantes. Entre garimpeiros, houve relatos de internações por malária grave, ataques prévios de malária e atrasos no tratamento após o início dos sintomas. Embora 73% (145) dos garimpeiros tenham relatado saber como a malária era transmitida, apenas 54% (107) usavam mosquiteiros ou repelentes. O uso de Artecom e cloroquina pelos garimpeiros não se destina ao tratamento completo, mas apenas ao alívio dos sintomas, permitindo o retorno às minas de ouro. Isso ressalta a importância da vigilância molecular para detectar a resistência antimalárica. Os indígenas são considerados uma população vulnerável à malária, e os garimpeiros promovem o aumento da malária nas Terras Indígenas de Roraima. Portanto, o acesso ao diagnóstico e tratamento em áreas indígenas invadidas por garimpeiros é imperativo para o enfrentamento dessa doença que assola as comunidades indígenas e ameaça a saúde pública em larga escala na meta de erradicar a malária no estado.

## Resumen

La malaria es un problema de salud pública y los casos diagnosticados en la capital de Roraima, Brasil, tienen el potencial de caracterizar la carga de la enfermedad en todo el estado. El objetivo de este estudio fue describir los aspectos epidemiológicos, clínicos y de laboratorio de los casos de malaria diagnosticados en Boa Vista. Para ello, se realizó un estudio descriptivo y transversal en dos unidades de salud del municipio, con personas diagnosticadas con malaria y que aceptaron responder el cuestionario. De 206 participantes, caracterizados como hombres, pardos y jóvenes, el 96% (198) reportó actividad minera ilegal. Entre los mineros, el 66% (131) procedían de otros estados de Brasil o de otros países. Las minas estaban ubicadas principalmente en el territorio Yanomami, en Roraima. La infección por *Plasmodium vivax* se produjo en el 74% (153) de los participantes. Entre los mineros, hubo relatos de hospitalizaciones por malaria grave, ataques previos de malaria y retrasos en el tratamiento tras de la aparición de los síntomas. Aunque el 73% (145) de los mineros informó saber cómo se transmitía la malaria, solo el 54% (107) usaba mosquiteros o repelentes. El uso de Artecom y cloroquina por parte de los mineros no está destinado a un tratamiento completo, sino al alivio de los síntomas para permitir el regreso a las minas de oro. Esto resalta la importancia de la vigilancia molecular para detectar la resistencia a los antipalúdicos. Los indígenas son considerados una población vulnerable a la malaria, y los mineros promueven el aumento de la malaria en las Tierras Indígenas de Roraima. Por lo tanto, el acceso al diagnóstico y tratamiento en zonas indígenas invadidas por mineros es imperativo para combatir esta enfermedad que asola a las comunidades indígenas y amenaza la salud pública a gran escala en el objetivo de erradicar la malaria en el estado.

## Introduction

Malaria is an acute febrile infectious disease, caused by the protozoan of the genus *Plasmodium*, transmitted by the female mosquito of the genus *Anopheles* ^1^ ^,^ ^2^. The disease is endemic in 85 countries (including the territory of French Guiana) located in tropical and subtropical regions of the world. In 2022, 249 million malaria cases and 608,000 deaths were estimated. In the Americas region, Venezuela, Brazil, and Colombia accounted for more than 73% of the 0.6 million cases that occurred in this region ^2^.

In Brazil, malaria is endemic in the Legal Amazon (Acre, Amapá, Amazonas, Maranhão, Mato Grosso, Pará, Rondônia, Roraima, and Tocantins states), which accounts for 99% of cases of the disease in the country. In 2021, 139,107 malaria cases and 61 deaths were recorded in the country ^3^. That same year, Roraima presented 26,005 autochthonous cases and 25 deaths from the disease ^4^.

Despite being a preventable and treatable disease with medicines available free of charge in the Brazilian Unified National Health System (SUS, acronym in Portuguese), morbidity and mortality data show that malaria is still an important public health problem. The disease negatively impacts both the health and livelihoods of people worldwide, especially the poorest and most vulnerable populations. Moreover, it poses challenges for its elimination, demanding successful strategies to achieve the goal of eliminating malaria transmission in Brazil by 2035 ^5^.

From 2010 to 2020, the period that precedes the decade of work for Brazil to achieve the elimination target, cases of malaria imported from Venezuela and mining in the Yanomami Indigenous area were identified as the factors responsible for the increase in cases of the disease in Roraima ^6^. The diagnosis of malaria occurred mainly in Boa Vista, the state capital. In this scenario, in addition to the clear impact on the municipal healthcare network, there is also the risk of outbreaks and epidemics in Boa Vista. In fact, the municipality shows a climate favorable to the main vectors of malaria, along with large rivers, streams, and lakes in the urban area of the city, characteristics that also favor the existence of breeding sites ^7^.

In 2022, according to data from the Malaria Epidemiological Surveillance Information System (SIVEP-Malaria), among the 26,204 autochthonous cases registered in Roraima, 12,010 were associated with mining activities, and 86% (10,329) of them were diagnosed/notified in Boa Vista, a Roraima municipality classified as low risk for malaria transmission (annual parasite incidence - API < 10 cases/1,000 inhabitants). In Boa Vista, 1% (206) were considered autochthonous and 99% were imported cases from other Roraima municipalities: Alto Alegre (75%), Mucajaí (17%), and Amajari (2%) or neighboring countries (mainly Venezuela and Guyana) ^8^.

The health units with the highest number of case notifications were the Cosme e Silva Emergency Service Unit and the Sayonara Maria Dantas Basic Health Unit, representing 31% (4,490) and 14% (2,075) of cases, respectively, accounting for almost 50% of the malaria cases notified in Boa Vista ^80^.

In the group of individuals seeking a malaria diagnosis in Boa Vista, it is possible to identify multiple factors that can characterize the epidemiological pattern of the malaria burden in the state of Roraima, which can support the formulation of effective public policies to control malaria disease following local reality ^9^.

Mining activity is allowed by law in Brazil. However, mining activities in the Amazon are mostly operated without a license, with mercury, within Indigenous Lands and Conservation Units, and without environmental recovery efforts. Such mining activities are considered illegal.

Given the representativeness of malaria cases diagnosed in Boa Vista, the objective of this work was to investigate where the infection occurs and the main activity of infected individuals via an epidemiological survey. Moreover, we investigated the clinical and laboratory aspects of malaria cases reported in the two units that concentrate the largest number of malaria notifications.

## Methodology

**Figure 1:**
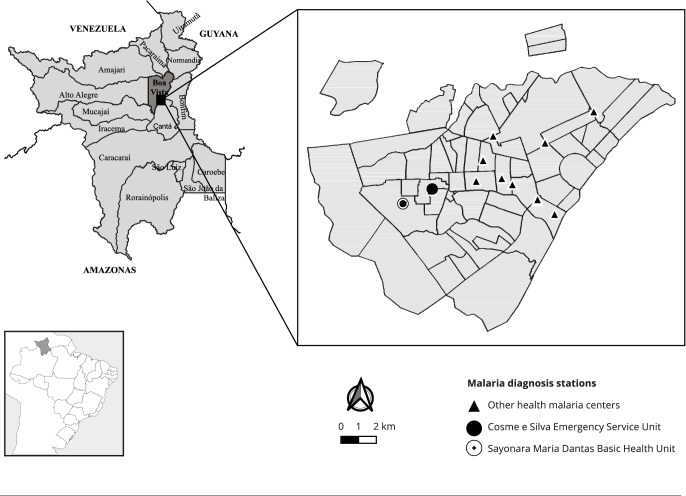
Location of the municipality of Boa Vista, state of Roraima, Brazil, showing the urban area, rivers networks and streams, and malaria diagnostic units.

Source: prepared by the authors.

The state of Roraima, located in the extreme north of Brazil, shares an international border with Venezuela and Guyana, as well as a national border with the states of Amazonas and Pará. In 2021, according to the Brazilian Institute of Geography and Statistics (IBGE, acronym in Portuguese) ^10^, Roraima held a population of 652,713 inhabitants. The state capital, Boa Vista, concentrates 67% of the population, with 436,591 inhabitants, and is bordered by the municipalities of Pacaraima, Normandia, Bonfim, Cantá, Mucajaí, Alto Alegre, and Amajari (Figure 1). It comprises 15 municipalities and 104,509.087km^2^ of its territory consists of Indigenous reservations. The Yanomami Indigenous Land is the largest Indigenous reserve in Brazil. The Special Indigenous Health District Yanomami (DSEI-Yanomami) in Roraima covers five municipalities: Alto Alegre, Amajari, Caracaraí, Iracema, and Mucajaí. All mining sites in Roraima are illegal and located within Indigenous Lands, and the largest of them are in Yanomami territories.

Boa Vista holds 15 malaria diagnostic centers located in the urban area of the city, six in hospital/urgency and emergency units; one in maternal and child hospital; and eight in primary health care (PHC) units. The study was conducted in two health units located in the west of Boa Vista: the Cosme e Silva Emergency Service Unit and the Sayonara Maria Dantas Basic Health Unit (Figure 1). These notification units were chosen due to holding the highest number of malaria notifications in Boa Vista, according to SIVEP-Malaria.

To achieve the proposed objective, a descriptive, cross-sectional study was conducted from December 2021 to June 2022, during the driest season of the year in Roraima, when malaria transmission is favored, consequently leading to an increase in diagnosis demand. Individuals over 18 years of age and diagnosed with malaria via thick blood smears were included in the study. Minors under 18 years of age, Indigenous people living in villages, those who were unable to read the informed consent form, and individuals who refused to sign it did not participate.

This work was approved by the Research Ethics Committee of the Federal University of Roraima (CEP/UFRR, acronym in Portuguese; opinion n. 3,920,373, issued on March 17, 2020). The CEP/UFRR allowed only the inclusion of non-village Indigenous people who speak Brazilian Portuguese and reside in Boa Vista. Additionally, the research project was demanded to be presented to the Kannu Kadan Indigenous Association for obtaining a letter of consent which was attached in the submission process to the CEP/UFRR.

Participants signed an informed consent form before responding a questionnaire containing questions related to the individual (gender, age, ethnicity, place of residence, main activity in the 15 days before the onset of symptoms, knowledge about the forms of malaria transmission, and prophylaxis) and the disease (symptoms, parasitic form causing the infection, and history of the disease). The questionnaires were typed and tabulated in the Excel software (https://products.office.com/).

The maps were created using the QGIS program version 3.28.10 (https://qgis.org/en/site/), and mining areas in Roraima were obtained from MapBiomas ^11^. Geopolitical limits of Brazil and Indigenous Lands were accessed on the IBGE website ^12^.

Statistical analysis was performed using the chi-square test with a significance level of 5%, using the BioEstat 5.33 program (https://mamiraua.org.br/downloads/programas/) ^13^.

## Results

According to SIVEP-Malaria, during the study period (December 2021 to June 2022), 7,379 malaria cases were notified in Boa Vista and 43% of them were diagnosed in the health units selected as strategic points for this research.

When analyzing participants’ responses about the main activity performed in the 15 days before the onset of symptoms, four activities were identified. Mining was the predominant activity (p < 0.0001) with 198 (96%) participants, followed by agriculture with 6 (3%), hunting/fishing with 1 (0.5%), and tourism with 1 (0.5%).

The age of the participants ranged from 18 to 67 years old, and the age groups 18 to 29 and 30 to 39 years old were prevalent in participants who reported mining activity (p < 0.0001). Of the total number of participants, men were more frequent than women (p < 0.0001), with 169 (82%) men. Additionally, mixed-race was the most frequent ethnicity, with 119 (58%) individuals (p < 0.0001). Fever (90.3%), headache (87.4%), chills (67.5%), abdominal pain (54.9%), and myalgia (53.4%) were found to be more predominant symptoms compared to sweating, diarrhea, dyspnea, low back pain, and no symptoms (p < 0.0001). In total, 202 participants (98%) reported at least one symptom. The same symptomology was described by the miners, being the only group with asymptomatic participants (four in total, accounting for 2%) (Table 1).

**Table 1:** Characterization of study subjects, according to the main activity performed 15 days before by age group, gender, ethnicity, symptoms, parasitic species, and epidemiological variables related to malaria. Boa Vista, state of Roraima, Brazil.

Characteristics	Illegal mining (n = 198)		Agriculture (n = 6)		Hunting/Fishing (n = 1)		Tourism (n = 1)		Total (n = 206)
	n	%	n	%	n	%	n	%	n	%
Age group (years)										
18-29	92	46.5	3	50.0	0	0.0	0	0.0	95	46.1
30-39	60	30.3	1	16.7	1	100.0	0	0.0	62	30.1
40-49	26	13.1	1	16.7	0	0.0	1	100.0	28	13.6
50-59	17	8.6	1	16.7	0	0.0	0	0.0	18	8.7
≥ 60	3	1.5	0	0.0	0	0.0	0	0.0	3	1.5
Gender										
Male	164	82.8	3	50.0	1	100.0	1	100.0	169	82.0
Female	34	17.2	3	50.0	0	0.0	0	0.0	37	18.0
Ethnicity										
White	32	16.2	2	33.3	0	0.0	0	0.0	34	16.5
Black	39	19.7	2	33.3	0	0.0	0	0.0	41	19.9
Asian	7	3.5	0	0.0	0	0.0	0	0.0	7	3.4
Mixed-race	115	58.1	2	33.3	1	100.0	1	100.0	119	57.8
Indigenous	5	2.5	0	0.0	0	0.0	0	0.0	5	2.4
Symptoms										
Fever	179	90.4	5	83.3	1	100.0	1	100.0	186	90.3
Headache	173	87.4	6	100.0	1	100.0	0	0.0	180	87.4
Chill	136	68.7	2	33.3	1	100.0	0	0.0	139	67.5
Abdominal pain	109	55.1	2	33.3	1	100.0	1	100.0	113	54.9
Myalgia	105	53.0	4	66.7	1	100.0	0	0.0	110	53.4
Nausea/Vomit	87	43.9	2	33.3	0	0.0	0	0.0	89	43.2
Sweating	78	39.4	2	33.3	1	100.0	0	0.0	81	39.3
Diarrhea	36	18.2	1	16.7	0	0.0	0	0.0	37	18.0
Dyspnea	27	13.6	0	0.0	0	0.0	0	0.0	27	13.1
Low back pain	8	4.0	0	0.0	0	0.0	0	0.0	8	3.9
No symptoms	4	2.0	0	0.0	0	0.0	0	0.0	4	1.9
Species										
*Plasmodium vivax*	147	74.2	4	66.7	1	100.0	1	100.0	153	74.3
*Plasmodium falciparum* + mixed malaria	51	25.8	2	33.3	0	0.0	0	0.0	53	25.7
Have you had malaria before?										
Yes	161	81.3	4	66.7	0	0.0	1	100.0	166	80.6
No	37	18.7	2	33.3	1	100.0	0	0.0	40	19.4
Have you ever been hospitalized?										
Yes	40	20.2	0	0.0	0	0.0	0	0.0	40	19.4
No	158	79.8	6	100.0	1	100.0	1	100.0	166	80.6
Do you know how malaria is transmitted?										
Yes	145	73.2	5	83.3	1	100.0	0	0.0	151	73.3
No	53	26.8	1	16.7	0	0.0	1	100.0	55	26.7
Prophylactic measures										
Mosquito nets	47	23.7	1	16.7	0	0.0	0	0.0	48	23.3
Repellent	60	30.3	1	16.7	0	0.0	0	0.0	61	29.6
Antimalarials	12	6.1	0	0.0	0	0.0	0	0.0	12	5.8
No prevention	79	39.9	4	66.7	1	100.0	1	100.0	85	41.3

Source: prepared by the authors.

Participants were most affected by *P. vivax* (p < 0.0001), which occurred in 153 (74.3%) individuals, followed by *P. falciparum* in 42 (20.4%), and mixed malaria (*P. falciparum* + *P. vivax*) in 11 (5.3%). Conversely, 24.7% of participants were affected by *P. falciparum* single or mixed infections.

Most participants 166 (81%) reported previous episodes of malaria (p < 0.0001). Moreover, most participants reported no previous hospitalization due to malaria (p < 0.0001). Hospitalizations were reported only in the miners’ group by 40 participants (20%).

The nonuse of prophylactic measures and the use of repellent were the most cited when asked about prophylactic measures (p < 0.0001). Moreover, only the miners’ group reported using antimalarials as a form of prophylaxis, with 14 (6%) reports (Table 1).

Concerning the timeliness of treatment, starting treatment within 96 hours and after 96 hours were more frequent than other periods, that is, up to 24 or 48 hours (p = 0.0024) and 57 (29.4%) miners started treatment after 96 hours since the onset of symptoms (Table 2).

**Table 2:** Characterization of the case series by the main activity conducted 15 days before the onset of symptoms, according to the period of initiation of treatment after the appearance of symptoms. Boa Vista, state of Roraima, Brazil.

Onset of symptoms/Treatment initiation	Illegal mining (n = 194)		Agriculture (n = 6)		Hunting/Fishing (n = 1)		Tourism (n = 1)		Total (n = 202)	
	n	%	n	%	n	%	n	%	n	%
Within 24 hours	32	16.5	0	0.0	0	0.0	0	0.0	32	15.8
Within 48 hours	43	22.2	1	16.7	0	0.0	0	0.0	44	21.8
Within 96 hours	62	32.0	4	66.7	1	100.0	0	0.0	67	33.2
After 96 hours	57	29.4	1	16.7	0	0.0	1	100.0	59	29.2

Source: prepared by the authors.

**Figure 2:**
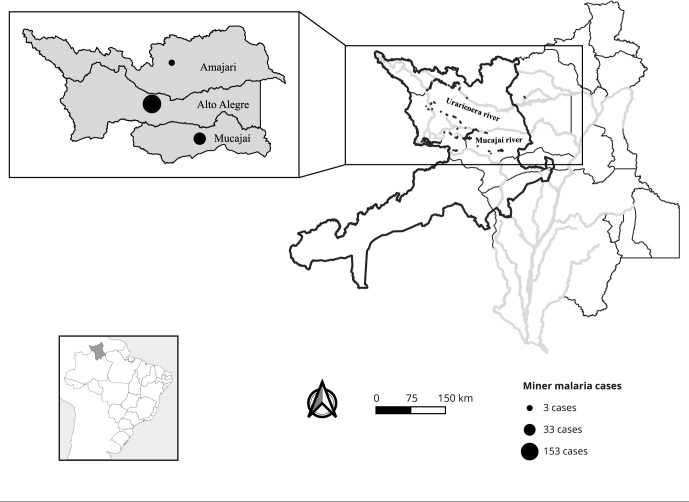
Municipalities of infection of participants who carried out mining activities 15 days before the onset of symptoms. Boa Vista, state of Roraima, Brazil.

Source: prepared by the authors.

Regarding miners, 95.5% (189/198) were infected in Indigenous territories located in three Roraima municipalities (Alto Alegre, Mucajaí, and Amajari): 81% (153/189) of them in illegal mines located along the Uraricoera River in Alto Alegre; 17.5% (33/189) in illegal mines located along the Mucajaí River and its tributary Couto de Magalhães in Mucajaí; and 1.5% (3/189) in mines also located along the Uraricoera River but in Amajari (Figure 2). In addition, imported cases were identified in patients who came from mining sites in Venezuela (4/198), Guyana (4/198), and state of Pará (1/198). Likewise, participants who reported agricultural activity, the probable location of infection was Alto Alegre (1/6) and Mucajaí (1/6), in addition to the municipalities of Cantá (1/6), Caracaraí (1/6), and Caroebe (2/6). The participant who reported hunting/fishing occupation, as well as with the other who reported tourism activity became infected in the municipality of Mucajaí (2/2).

**Figure 3:**
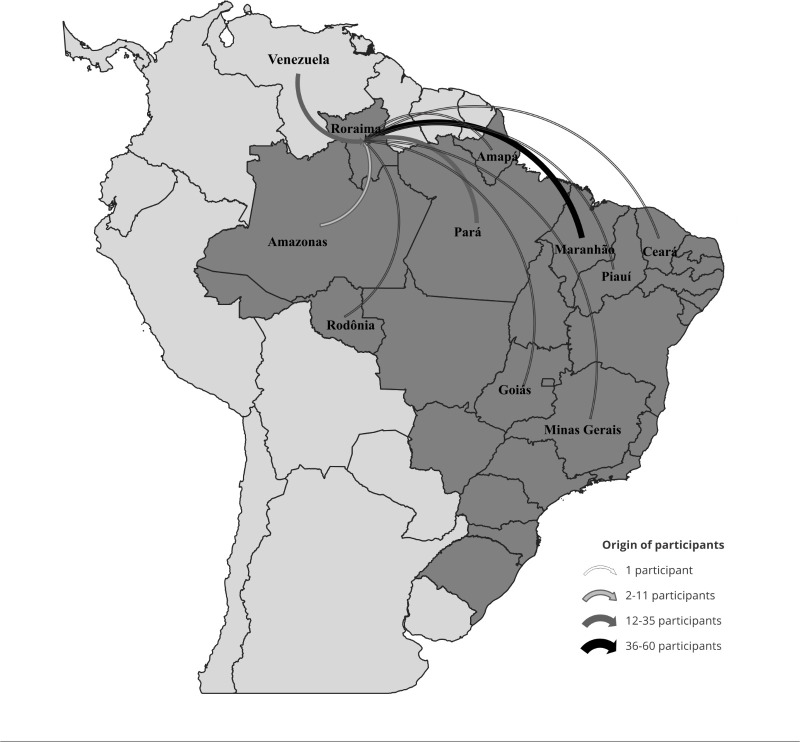
Origin of study participants who carried out illegal mining activity 15 days before diagnosis. Boa Vista, state of Roraima, Brazil.

Source: prepared by the authors.

Most participants with mining activity came from Roraima 67 (34%) and Maranhão 60 (33%), followed by Pará 33 (17%), Venezuela 22 (11%), and Amazonas 7 (3.5%) (p < 0.000). The states of Piauí (2), Amapá (2), and Ceará (2) were the place of residence of 1% of the participants, while Goiás, Minas Gerais, and Rondônia contributed each one with one participant (0.5%) (Figure 3).

Concerning participants who performed agricultural activities, around 33% (2) came from Roraima and the remaining were from Pará, Amazonas, Bahia, and Venezuela. The participant who performed hunting/fishing occupation was from Pará and the participant who reported working with tourism was from Piauí.

## Discussion

This is a descriptive study. We highlight that the selected units are representative of the malaria-diagnosed cases in Roraima. Our casuistic comprises cases of passive surveillance/search. Therefore, we may be underestimating the number of cases since individuals who perform self-treatment in mining, without seeking health facilities, could only be identified by active search in mining areas in Yanomami lands. However, this active search is hindered by the location of the mines in remote forest areas whose access is often via clandestine take-off and landing strips, besides the risks to the researchers’ lives in these conflict areas.

Identifying the place of infection is essential to guide the planning of malaria prevention and control actions. In Brazil, cases of malaria in urban and rural areas and settlements have decreased since 2017, contrasting with cases in miners and Indigenous people, which increased around 257% and 62%, respectively ^6^ ^,^ ^14^.

In this study, 96% of the participants reported mining activity at least 15 days before the onset of symptoms, strongly suggesting that they were infected in Indigenous Lands where all Roraima illegal mines are located.

The presence of miners in the Yanomami Indigenous territories contributed to the increased number of malaria cases in Roraima in recent years. Even in 2020, during the COVID-19 pandemic, there was a 44% increase in autochthonous malaria cases in Roraima and in the Yanomami Indigenous Land, which corresponds to a 77% increase when compared to 2019, disregarding the increased number of hospitalizations and deaths. In this same period, a 30% increase in mining activity was also noted in the Yanomami Indigenous area ^6^. The mines are very close to the Indigenous communities. The Uraricoera River concentrated 52% of the entire area degraded by mining; gold exploration takes place in the Waikás, Araçá, and Korekorema Indigenous communities. The mines along the Mucajaí River (and its tributary Couto de Magalhães) represent almost 25% of the total mining activities; gold exploration occurs in the borders of the Kayanau and Papiu Indigenous communities ^15^.

Indigenous reserves are considered the most preserved areas and are responsible for forest conservation ^16^. The mining activity in Yanomami Indigenous Land has multiple impacts on traditional communities and the ecosystem ^16^. Disintrusion is necessary, but it is not a simple or low-cost process. In this context, a robust political, economic, and public security project must be developed. Evidence of the presence of organized crime (called *narco* mining) makes the process even more difficult ^16^ ^,^ ^17^, as well as health actions to control malaria in these areas.

Mining is a major challenge for the elimination of malaria over the next decade in Brazil. In addition to requiring health and environmental interventions, there is a need to formulate control policies that include the specificity of the garimpeiro population, despite the illegality of mining activity ^6^ ^,^ ^14^ ^,^ ^18^ since controlling malaria in this population can reduce the impact of malaria on Indigenous people.

Miners can increase malaria transmission via three main factors: (a) modification of the environment, with the use of hoses and combustion engines to extract the sediment, forming pools of water that serve as breeding grounds, enhancing the reproduction of the mosquito vector, especially *A. darlingi*; (b) more exposition to mosquitoes by work outdoors for long periods a day and sleep in tents with incomplete walls; and (c) the intense mobility between the mining areas and their place of residence or other areas, promoting transmission and the occurrence of outbreaks ^19^ ^,^ ^20^ ^,^ ^21^ ^,^ ^22^.

The number of women in mining activity, although smaller, should also be considered for strategies to eliminate malaria, especially due to the possibility of pregnancy, as severe malaria disease, when not treated promptly, can lead to conditions such as, maternal and fetal anemia; premature birth; low birth weight and fetal problems; and maternal and neonatal death ^23^ ^,^ ^24^ ^,^ ^25^.

Fever, followed by headache, was the most frequent symptom among study participants. Fever remains the main symptom for health education strategies on the identification of malaria symptoms. Therefore, the advice to seek a health unit if one is feeling feverish and has been in an area at risk for malaria transmission continues to be an important guideline for timely diagnosis and treatment. We highlight that cultural differences must be considered so that this strategy is effective, especially in territories with multi-ethnic borders ^26^ ^,^ ^27^.

In the group of miners, 81% (161) had previous malaria attacks and 2% (4) had no symptoms at the time of diagnosis. It was not possible to define whether previous episodes of malaria were related to relapse, recrudescence, or reinfection. Furthermore, miners reported that soon after the diagnosis and treatment for malaria, they returned to the mine, often on the same day, thus being constantly exposed to areas where malaria is transmitted.

The silent condition of asymptomatic carriers represents a challenge to the elimination of malaria ^25^ ^,^ ^28^; therefore, surveillance must also track the movement of human carriers. The asymptomatic participants engaged in this study were relatives of the patients who had the malaria diagnosis at Boa Vista. Since they also came from mining areas, the malaria microscopic test was performed.

Regarding the parasitic form, 53 cases of infection by *P. falciparum* and *P. falciparum* + *P. vivax* (mixed malaria) were identified, of which 51 were in miners. In the Americas region, *P. vivax* malaria represents 75% of cases ^22^. However, in mining areas, *P. falciparum* appears to be more frequent than in other ones ^18^ ^,^ ^28^. Notably, among the 11 cases of mixed *P.falciparum*/*P.vivax* infections, 10 were in miners. In this context, updating microscopists or adopting more precise diagnostic procedures is crucial for adequate treatment. If mixed infections are misdiagnosed as a *P. vivax* monoinfection and treatment is prescribed only for this parasite, the increase in *P. falciparum* parasitemia may evolve into severe malaria ^29^.

For miners, absenteeism due to malaria causes a major financial impact. Thus, self-medication and the use of sub-doses of medication as a form of prophylaxis or quick relief of symptoms are common in this group ^21^ ^,^ ^30^. In the national malaria treatment protocol, chloroquine is the first-line treatment for uncomplicated malaria caused by *P. vivax*. A combination of chloroquine is prescribed for three days (10mg/kg on day 1 and 7.5mg/kg on days 2 and 3) and primaquine (0.5mg/kg/day, for seven days), aiming to cure both the blood form and the hepatic form (radical cure), respectively, thus avoiding relapse due to hypnozoites ^31^. However, the medication used by the miners who participated in this study was Artecon, which could be purchased at the mine itself. This drug is not recommended by local pharmaceutical authorities and the World Health Organization and it is illegal in Brazil, French Guiana, and neighboring countries ^28^ ^,^ ^32^.

In this way, the increase in the number of infections, in addition to the emergence of resistance to antimalarials could occur, reinforcing the need for molecular surveillance via antimalarial resistance markers in mining areas, especially in the case of artemisinin ^18^ ^,^ ^33^ ^,^ ^34^ ^,^ ^35^.

The Brazilian National Malaria Control Program considers treatment with antimalarials to be opportune when it is conducted within 48 hours from the onset of symptoms for autochthonous cases and within 96 hours for imported cases ^5^. However, most miners reported starting treatment after 96 hours or more. In this study, in the group of miners, 29% (57) were diagnosed after 96 hours.

In this scenario, it is important to introduce diagnosis and treatment in mining to interrupt the transmission and, consequently, reduce the burden of malaria in Indigenous areas. However, in Roraima, given the illegality of mining activity together with the *narco* mining ^17^, healthcare teams are unsafe to perform diagnosis and treatment in mining areas.

The Malakit Project was a successful and innovative strategy with an impact on the reduction of malaria cases in miner populations in French Guiana and Suriname. This intervention proposed to provide miners with training on malaria, kits for self-diagnosis via rapid testing, and self-treatment ^32^ ^,^ ^36^. In Roraima, this strategy could be adopted at health units diagnosing malaria in miners to overcome lack of security in conflict areas.

Notably, 66% of the miners in this study came from other Brazilian areas, including those of the extra-Amazonian region, or Venezuela. The mobility of miners is a challenge to eliminating malaria since infected people cross borders and increase the possibility of reintroducing malaria into areas where malaria has been reduced or eliminated, as well as spreading malaria parasites ^34^ ^,^ ^35^ ^,^ ^37^.

## Conclusion

The snapshot of malaria diagnosed in Boa Vista shows that the burden of malaria transmission in the state occurs in the mines located in the Yanomami Indigenous Land. The presence of miners increases the number of malaria cases in Roraima, including in its Indigenous reserve.

The disintrusion of miners from Yanomami territories is urgently needed and requires a robust and complex long-term government project. Meanwhile, public health in Brazil can conduct strategies to control malaria among miners to protect the Indigenous peoples and decrease the transmission in Roraima. These strategies can aid reducing malaria cases in the Yanomami Indigenous population and, consequently, achieving the goal of eliminating malaria by 2035. To this end, surveillance must be also implemented with cooperative policies between municipalities and federal units in Brazil and neighboring countries to detect the mobility routes of garimpeiro populations.

## References

[1] Departamento de Articulação Estratégica de Vigilância em Saúde e
Ambiente, Secretaria de Vigilância em Saúde e Ambiente, Ministério da
Saúde (2023). Guia de vigilância em saúde..

[2] World Health Organization (2023). World malaria report 2023.

[3] Secretaria de Vigilância em Saúde e Ambiente, Ministério da
Saúde (2021). Malária.. Boletim Epidemiólogico.

[4] Coordenação Geral de Vigilância em Saúde, Secretaria de Estado da
Saúde Relatório anual epidemiológico de Roraima, 2021..

[5] Departamento de Imunização e Doenças Transmissíveis.Secretaria de
Vigilância em Saúde.Ministério da Saúde (2022). Elimina Malária Brasil: Plano Nacional de Eliminação da Malária.

[6] de Aguiar Barros J, Granja F, Pequeno P, Marchesini P, Ferreira da Cruz MF (2022). Gold miners augment malaria transmission in indigenous
territories of Roraima state, Brazil.. Malar J.

[7] Louzada J, de Almeida NCV, de Araujo JLP, Silva J, Carvalho TM, Escalante AA (2020). The impact of imported malaria by gold miners in Roraima
characterizing the spatial dynamics of autochthonous and imported malaria in
an urban region of Boa Vista. Mem Inst Oswaldo Cruz.

[8] Ministério da Saúde Dados para cidadão a partir da fonte de dados do Sivep-Malária, Sinan e
E-SUS-VS, para notificações do Brasil de 2007 a 2023..

[9] Barata RCB (1995). Malária no Brasil panorama epidemiológico na última
década. Cad Saúde Pública.

[10] Instituto Brasileiro de Geografia e Estatística Cidades e Estados. Roraima..

[11] MapBiomas Collection 7.0 of the 2021 Annual Series of Land Use and Coverage Maps
of Brazil..

[12] Instituto Brasileiro de Geografia e Estatística Cartas de imagem, camadas vetoriais de limites geopolíticos e Terras
Indígenas do Brasil..

[13] Ayres M, Ayres M, Ayres DL, Santos AAS (2007). BioEstat 5.3: aplicações estatísticas nas áreas das Ciências
Biomédicas..

[14] Castro MC, Peterka C (2023). Malaria is increasing in Indigenous and artisanal mining areas in
the Brazilian Amazon. Nat Med.

[15] Hutukara Associação Yanomami; Associação Wanasseduume
Ye'kwana (2021). Cicatrizes na floresta: evolução do garimpo ilegal na TI Yanomami em
2020..

[16] Basta PC (2023). Gold mining in the Amazon the origin of the Yanomami health
crisis. Cad Saúde Pública.

[17] Chagas RP (2011). O "narcogarimpo" na terra indígena yanomami.

[18] Shanks GD, Wongsrichanalai C (2021). Mining-associated malaria epidemics. Am J Trop Med Hyg.

[19] Arisco NJ, Peterka C, Castro MC (2021). Cross-border malaria in Northern Brazil. Malar J.

[20] Schwartz FW, Lee S, Darrah TH (2021). A review of the scope of artisanal and small-scale mining
worldwide, poverty, and the associated health impacts. Geohealth.

[21] Murta FLG, Marques LLG, Santos APC, Batista TSB, Mendes MO, Silva ED (2021). Perceptions about malaria among Brazilian gold miners in an
Amazonian border area perspectives for malaria elimination
strategies. Malar J.

[22] Recht J, Siqueira AM, Monteiro WM, Herrera SM, Herrera S, Lacerda MVG (2017). Malaria in Brazil, Colombia, Peru and Venezuela current
challenges in malaria control and elimination. Malar J.

[23] Luz TC, Miranda ES, Freitas LF, Osório-de-Castro CG (2013). Prescriptions for uncomplicated malaria treatment among pregnant
women in the Brazilian Amazon evidences from the Mafalda
Project. Rev Bras Epidemiol.

[24] Cardona-Arias JA (2022). Systematic review of mixed studies on malaria in pregnancy
individual, cultural and socioeconomic determinants of its treatment and
prevention. Trop Med Infect Dis.

[25] Mendes LMC, Gomes-Sponholz F, Monteiro JCS, Pinheiro AKB, Barbosa NG (2022). Women who live in mining on the French-Brazilian border daily
challenges. Rev Bras Enferm.

[26] Silva RSU, Carvalho FT, Santos AB, Ribeiro ES, Cordeiro KM, Neiva GIBM (2012). Malária no Município de Cruzeiro do Sul, Estado do Acre, Brasil
aspectos epidemiológicos, clínicos e laboratoriais. Rev Pan-Amazônica Saúde.

[27] Bria YP, Yeh CH, Bedingfield S (2022). 2022 International Joint Conference on Neural Networks.

[28] Gaillet M, Musset L, Cropet C, Djossou F, Mallard A, Odonne G (2023). Determination of different social groups' level of knowledge
about malaria in a multicultural Amazonian cross-border
context. BMC Public Health.

[29] Kotepui M, Kotepui KU, De Jesus Milanez G, Masangkay FR (2020). Plasmodium spp. mixed infection leading to severe malaria: a
systematic review and meta-analysis.. Sci Rep.

[30] Lima ISF, Duarte EC (2017). Factors associated with timely treatment of malaria in the
Brazilian Amazon a 10-year population-based study. Rev Panam Salud Pública.

[31] Departamento de Imunização e Doenças Transmissíveis, Secretaria de
Vigilância em Saúde, Ministério da Saúde (2021). Guia de tratamento da malária no Brasil..

[32] Longchamps C, Galindo MS, Lambert Y, Sanna A, Mutricy L, Garancher L (2022). Impact of Malakit intervention on perceptions, knowledge,
attitudes, and practices related to malaria among workers in clandestine
gold mines in French Guiana results of multicentric cross-sectional surveys
over time. Malar J.

[33] Evans L, Coignez V, Barojas A, Bempong D, Bradby S, Dijiba Y (2012). Quality of anti-malarials collected in the private and informal
sectors in Guyana and Suriname. Malar J.

[34] Laporta GZ, Grillet ME, Rodovalho SR, Massad E, Sallum MAM (2022). Reaching the malaria elimination goal in Brazil a spatial
analysis and time-series study. Infect Dis Poverty.

[35] Grillet ME, Moreno JE, Hernández-Villena JV, Vincenti-González MF, Noya O, Tami A (2021). Malaria in Southern Venezuela the hottest hotspot in Latin
America. PLoS Negl Trop Dis.

[36] Douine M, Sanna A, Galindo M, Musset L, Pommier de Santi V.Marchesini P (2018). Malakit an innovative pilot project to self-diagnose and
self-treat malaria among illegal gold miners in the Guiana
Shield. Malar J.

[37] De Salazar PM, Cox H, Imhoff H, Alexandre JSF, Buckee CO (2021). The association between gold mining and malaria in Guyana a
statistical inference and time-series analysis. Lancet Planet Health.

